# 2094 Validation of a set of “healthcare trust” scales for women seeking substance abuse treatment in community-based settings

**DOI:** 10.1017/cts.2018.269

**Published:** 2018-11-21

**Authors:** Joshua Cockroft, Deondria Matlock, Susie Adams

**Affiliations:** 1 Vanderbilt University Medical Center; 2 The Next Door, Inc; 3 Vanderbilt University School of Nursing

## Abstract

OBJECTIVES/SPECIFIC AIMS: To validate previously published psychometric scales capturing interpersonal or healthcare-related trust in a target population of women with a history of substance use disorder seeking substance abuse treatment in a community-based setting. METHODS/STUDY POPULATION: Participants are enrolled at The Next Door, Inc. (TND) and Renewal House (RH), 2 community agencies in metropolitan Nashville that provide substance abuse treatment and post-incarceration re-entry services for women with a history of substance use disorder. We will enroll 300 participants to provide sufficient power for statistical psychometric validation. Inclusion criteria include adult women with self-identified history of substance use disorder seeking substance abuse treatment within seven days of initiation of inpatient residential or intensive outpatient treatment at TND or RH. Participants complete a one-time online survey comprising a demographics questionnaire, Rotter Interpersonal Trust Scale, Wake Forest Trust in Physician Scale, Revised Health Care System Distrust Scale, 5-item RAND Social Desirability Scale, and Adverse Childhood Events Survey. Participants then individually participate in a modified protocol of the “Trust Game.” Predictor variables for multivariate analysis collected include age, race/ethnicity, gender identification, number of days in current treatment, number of prior substance abuse treatment programs, and number of adverse childhood events. RESULTS/ANTICIPATED RESULTS: Each individual scale will be assessed for item analysis, factor analysis, construct validity, content validity, and reliability and compared with general population sample values published in the literature. We will use multivariate analysis to determine the impact of potential predictor variables on specific types of interpersonal or healthcare-related trust. We anticipate having preliminary results to present in April. DISCUSSION/SIGNIFICANCE OF IMPACT: Women who seek substance abuse treatment in the community face unique challenges compared to their male counterparts, including higher rates of prior interpersonal trauma, co-occurring psychiatric diagnoses, and more serious physical health problems. Characteristics such as these highlight the need for regular healthcare engagement in the setting of an increased risk of decreased interpersonal or healthcare-related trust. Prior qualitative research demonstrates that trust building is seen as an essential component of care in ongoing substance abuse treatment for women in this population. Validation of psychometric healthcare-related trust scales in a population of women seeking substance abuse treatment in a community based setting will provide a framework for future quantitative inquiry into the impact of healthcare-related trust on health outcomes, healthcare engagement, and treatment retention for this target population. Similarly, it will also facilitate inquiry into the effectiveness of specific treatment programs or interventions on improving therapeutic trust building.

